# Successful Full Lactation Achieved by Mothers of Preterm Infants Using Exclusive Pumping

**DOI:** 10.3389/fped.2020.00191

**Published:** 2020-04-24

**Authors:** Xifang Ru, Xiaofang Huang, Qi Feng

**Affiliations:** Department of Pediatrics, Peking University First Hospital, Beijing, China

**Keywords:** human milk production, breastfeeding, breast pump, lactation, prematurity, nutrition

## Abstract

**Background:** Some mothers of preterm infants suffer from difficulties in initiating and maintaining adequate milk production. This study was designed to investigate the milk production in mothers of preterm infants using hospital-grade breast pumps and to study the nutrient content of their milk.

**Methods:** This was an observational prospective study. Mothers of preterm infants with gestational age < 32 weeks or birth weight < 1,500 g who were admitted to our hospital from August 2016 to December 2017 were recruited. A pumping diary and a questionnaire were completed by mothers (*n* = 30). Milk samples from before, during, and after each pumping session on days 7 and 14 postpartum were analyzed.

**Results:** The median time to onset of lactogenesis II was 75.4 h. Mean milk output increased gradually, meeting key thresholds of 350 g/d on day 6, 500 g/d on day 8, and close to 750 g/d on day 14. Then, all mean milk outputs were over 750 g/d. The mean milk output exceeded the mean feeding volume on days 7, 14, 21, 28, 35, and 42. Mothers using hospital-grade breast pumps had more cumulative milk production compared with mothers using hand expression. The milk yield on days 7 and 14 were positively correlated to that on days 21, 28, 35, and 42. Milk outputs on days 7, 14, and 42 of mothers with a pumping frequency of ≥ 6 times/d were greater than mothers with a pumping frequency of < 6 times/d. Threshold values for milk output on day 7 (cut-off, 406.8 g/d; sensitivity, 68.2%; specificity, 100%) and day 14 (cut-off, 518.0 g/d; sensitivity, 81.8%; specificity, 100%) were identified as predicting a milk output of more than 750 g/d on day 42. Fat and energy levels were higher in hind-milk than fore- or mixed-milk on days 7 and 14. Those who pumped ≥ 6 times/d had higher levels of fat, carbohydrate, and energy in their milk on day 7.

**Conclusion:** Most preterm infants' mothers using a hospital-grade pump with a pumping frequency of ≥ 6 times/d can reach full lactation successfully. Nutrient analysis of the human milk should be performed.

## Introduction

Human milk, especially mother's own milk, is highly recommended for preterm infants due to the beneficial effects it has on their development and health (1). Feeding with mother's own milk is now considered to be the best practice for preterm infants to reduce the risk of necrotizing enterocolitis and is believed to have a dose-dependent effect on neurodevelopmental improvement (2).

Unlike term infants, most preterm infants are unable to feed directly at the breast due to immaturity, illness, maternal-infant separation, etc. Under such circumstances, mothers are breast pump-dependent and often suffer from difficulties in initiating and maintaining adequate milk production. However, many mothers feel that the provision of milk is the only positive contribution they can make to the growth of their babies in this critical period, and failing to do so increases maternal anxiety (3). Therefore, establishing lactation (in the absence of direct breastfeeding) and developing an adequate expressing regime (method and schedule) are essential for the well-being of both mother and infant.

For mothers of preterm infants globally, insufficient milk volume remains a concern (4). Studies have demonstrated that the first 2 weeks after birth are a critical period for the initiation and development of lactation, during which mammary epithelial cells appear to undergo programming processes that regulate long-term milk synthesis (5, 6).

There is strong evidence that, for pump-dependent mothers, using a hospital-grade electric breast pump results in greater milk output than other expression methods (6–8). Many different recommendations are available in the literature regarding the use of a breast pump and the development of expression schedules (frequency) for the expressing mother. Current recommendations suggest that breast pumping should be performed with the same frequency as breastfeeding of a full term infant, i.e., every 3 h, night break of no more than 5 h, milk expression at least five times each day, and cumulative time at the breast pump of more than 100 min per day (9, 10).

However, these are generalized recommendations with no regard to the individual differences between mothers. In order to make individualized recommendations and to maximize milk output, it is necessary to identify the major determinants of milk production and to understand how these vary between individual mothers. Although it is challenging to establish milk supply in mothers of very-low-birth-weight (VLBW) infants early in the postpartum period, its importance should be emphasized.

The primary aim of this study was to investigate the initiation of lactation and the milk production with the help of hospital-grade breast pumps in mothers who were separated from their preterm infants in our neonatal intensive care unit (NICU). The secondary aim was to study the effect of breast pumping frequency on the volume and nutrient content of expressed milk.

## Materials and Methods

### Study Setting

Mothers who planned on breastfeeding and their preterm infants who were admitted to our NICU from August 2016 to December 2017 were involved in our study.

This was an observational prospective study carried out in the NICU of our hospital. The hospital was a Baby-Friendly Hospital, lactation services, such as breastfeeding knowledge and skills education, were available from obstetricians, obstetric nurses, and NICU team during hospitalization and the postpartum period. In our hospital, mothers were unable to stay with their preterm babies in the NICU, and no donor milk bank was available. The only way for mothers to breastfeed their preterm infants was to provide their own expressed milk. The obstetric nurses instructed each mother on the technique for milk expression through educational video, verbal instruction, and hands-on support for first milk expression. All participating mothers received standardized instructions on breast pumping by NICU doctors specializing in breast milk consultation several hours after delivery. Key recommendations, including starting pumping as soon as possible after delivery (target was within 6 h after delivery) and pumping every 2–3 h with no more than a 5-h break during the night, were communicated to the mothers. All mothers were seen daily by doctors and nurses during hospitalization to review milk expression techniques and to answer questions. Our breastfeeding hotline was open day and night to answer breastfeeding related inquiries.

A hospital-grade electric breast pump (Symphony, Medela AG, Switzerland) was provided to each mother immediately after enrollment. The pump was provided free of charge for the first 42 days postpartum, and all related disposable devices and milk containers were provided, whether in the hospital or at home. Mothers had the option to rent the pump if it was still needed after the 42-day period. Detailed information on breastfeeding was distributed and instructions on pump usage were given by the researchers. Before the onset of lactogenesis II (OOL-II), the initiation program (initiation breast pump suction patterns, I-BPSP) was selected for each pumping session. Once the OOL-II occurred as determined by the mother expressing a volume of 20 mL for three consecutive pumping sessions, the pumping program was switched to the maintenance program (standard breast pump suction patterns, S-BPSP). Mothers continued pumping for an additional 2 min after the last droplets of milk were noted to ensure that breasts were sufficiently drained. Mothers were encouraged to express their breast milk using the maximum comfortable vacuum as determined according to the mothers' own preferences. The maximum comfortable vacuum was determined by slowly increasing the vacuum until it felt slightly uncomfortable (not painful) and then decreasing it slightly. The selected pressure was then maintained over the course of the study.

### Inclusion and Exclusion Criteria

The inclusion criteria for enrollment included: (a) infants' gestational age (GA) < 32 weeks or birth weight < 1,500 g; (b) infants admitted to the NICU within the first 12 h of birth; (c) mothers over 18 years old; (d) mothers willing to breastfeed; (e) mothers joining our study within 24 h postpartum. The exclusion criteria were: (a) preterm infants who were very critical with unstable vital signs; (b) preterm infants or their mothers who had breastfeeding contraindications; (c) hospitalization for < 2 weeks or discharged against medical advice.

### Measurements

Daily milk output was defined as the cumulative milk volume from both left and right breasts from midnight to midnight. Milk output was measured by an electronic digital scale to the nearest 0.1 g. The time to the OOL-II was defined as the time point whenever milk output ≥ 20 g for three consecutive pumping sessions.

A dedicated human milk pumping diary recording pumping frequency and duration and milk output for each pumping session was completed for this study over the first 14 days, and on the 21st, 28th, 35th, and 42nd days postpartum. All mothers completed a questionnaire 5–7 days after enrollment.

Three characteristics of the OOL-II were measured: time to establish OOL-II; number of pumping sessions from birth to the OOL-II; and cumulative duration of pumping until the OOL-II.

Milk samples were collected from the mothers who were enrolled from September 2017 to December 2017. Milk (~1 mL) was taken from each breast before (fore), during (mixed), and after (hind) each pumping session on days 7 and 14 postpartum. Milk samples were collected in plastic tubes (with scale lines), stored at −18°C, and transferred to the hospital as soon as possible. Consultations on milk pumping and sampling were provided by the researchers around the clock. Phone call reminders for sample collections were made on days 6 and 13, 1 day before each milk sampling.

A human milk analyzer (HMA, Miris®, Sweden) was used for macronutrient analysis, as per the manufacturer's instructions. Briefly, the frozen milk samples were thawed at 40°C, stirred, and homogenized with ~2 mL (mixture of 1 mL from each breast) of milk used per analysis.

### Data Collection

Data were collected and collated by designated well-trained researchers. The dedicated human milk pumping diaries were requested. Milk nutritional data were recorded in the data system in a timely manner. Data were kept secure under the rigorous management of our hospital. Original data collected from the diaries were archived for quality control in a well-protected data system. The data were not permitted to be utilized for commercial purposes.

### Data Analysis

Data analysis was performed using Statistical Package for the Social Sciences (SPSS) software for Windows version 20.0 (SPSS Inc, Chicago, IL, USA). Data with normal distribution were expressed as mean ± standard deviation (SD). Comparison of the various continuous parameters was performed using univariate analysis of variance and *t*-test. Non-normal data were expressed as *M* (*P*_25_*, P*_75_) and compared with non-parametric test. Correlation analysis was performed using Pearson correlation. Receiver operating characteristic (ROC) curves were drawn using SPSS to explore the predictive value of milk output of more than 750 g or 1,000 g on day 42. A *P*-value of < 0.05 was considered to be statistically significant.

## Results

### Characteristics of the Study Population and Pumping Sessions

Initially, 33 mothers of preterm infants who met the inclusion criteria were included. However, three were later excluded from our study, as one found it difficult to keep up with the pumping and two did not keep records as we requested. The characteristics of the study population were shown in Table 1. The mean age of the mothers was 32.9 ± 4.5 years, 19 underwent cesarean sections, 9 were multiparous, and 7 had multiple births. The mean GA was 29.7 ± 1.8 weeks, and the mean birth weight was 1235.7 ± 260.8 g. The GA of 22 (59.5%) infants was <= 30 weeks, and 28 (75.8%) infants were VLBW infants (<1,500 g). None of the mothers smoked or drank during pregnancy or lactation, and 14 mothers had ≥ 2 maternal risk factors for preterm birth.

**Table 1:** The characteristics of the study population, pumping performance and OOL-II related information.

**Characteristics**	***n***	**Mean ± SD, *M* (*P_**25**_, P_**75**_*) or *n* (%)**
Mothers' age (years)	30	32.9 ± 4.5
Cesarean delivery	30	19 (63.3)
After IVF-ET	30	7 (23.3)
Multipara	30	9 (30.0)
Gestational ages (weeks)	30	29.7 ± 1.8
Birth weight (g)	37	1235.7 ± 260.8
Multiple birth	30	7 (23.3)
Pre-pregnancy BMI (kg/m^2^)	30	24.1 ± 4.5
Weight gain during pregnancy (kg)	30	8.0 ± 4.2
Drinking alcohol or smoking during pregnancy or lactation	30	0 (0.0)
Maternal risk factor for preterm birth	30	28 (93.3)
Premature rupture of membranes	30	5 (16.7)
Pre-eclampsia/eclampsia	30	3 (10.0)
Maternal diabetes	30	1 (3.3)
Infection	30	3 (10.0)
Placenta previa, vaginal bleeding	30	1 (3.3)
Placental abruption	30	1 (3.3)
≥ 2 maternal risk factors	30	14 (46.7)
Breast inflamation or surgery	30	0 (0.0)
Gestational ages ≤ 30 weeks	37	22 (59.5)
Birth weight <1,500 g	37	28 (75.8)
First pumping postpartum (h)	30	7.6 (4.6, 10.3)
Time to establish OOL-II		
After birth (h)	30	75.4 (60.7, 83.9)
After 1st pumping (h)	30	65.8 (56.6, 76.5)
Number of pumping sessions from birth to the OOL-II (times)	30	16.0 (13.8, 21.0)
Cumulative duration of pumping until the OOL-II (h)	30	4.0 (3.4, 5.3)
Breast pumping frequency (times/d, within 42 d after delivery)	30	6.7 ± 0.7

*IVF-ET, in vitro fertilization and embryo transfer; BMI, body mass index; OOL-II, onset of lactogenesis II*.

### Onset of Lactogenesis II

The median time from delivery to starting pumping was 7.6 h. The median time from delivery to OOL-II was 75.4 (60.7, 83.9) h (Table 1). Of these, 40% (12/30) of mothers reached the OOL-II within the recommended 72 h, with a mean time of 57.5 h (ranging from 44.6 to 72.0 h), while a delayed onset of lactogenesis II (DOL-II) occurred in 60% (18/30) of the participants. Moreover, two mothers failed to reach the OOL-II throughout the 42-day study period.

### Pumping Frequency

**Figure 1:**
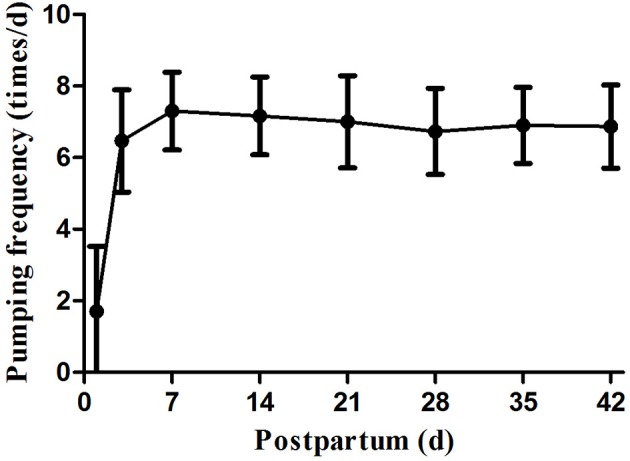
Pumping frequency during study period.

The mean frequency of pumping sessions within 42 days after delivery was 6.7 times/d. In the first 7 days postpartum, maternal pumping frequency gradually increased; after day 7, pumping frequency remained relatively stable at ~6–8 times/d (Figure 1).

### Daily Human Milk Output

**Figure 2:**
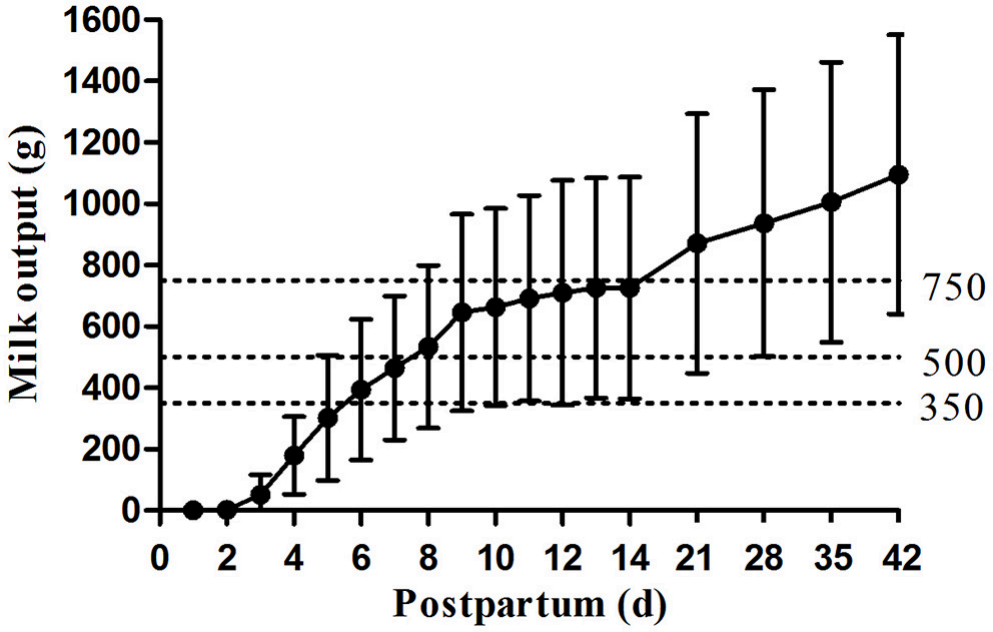
Daily maternal milk output in 1st 42 days postpartum.

Daily Milk output of preterm infants' mothers increased steadily from the OOL-II establishment until 6 weeks postpartum, with a particularly rapid increase from day 7 to day 28 (Figure 2). Large variations in milk output were found among the mothers throughout the study, and the disparity became more apparent as time went by, e.g., 107.3 g/d vs. 1,850.0 g/d on day 42. Mean milk output increased as the study progressed, meeting key thresholds of 350g/d on day 6, 500 g/d on day 8, and close to 750 g/d on day 14. Then, all mean milk outputs were over 750 g/d. The percentage of daily milk output ≥ 350 g/d, ≥ 500 g/d, and ≥ 750 g/d are shown in Table 2. On day 42, only 2 mothers (6.7%) did not reach 350 g/d or 500 g/d, and 8 mothers (26.7%) did not reach 750 g/d.

**Table 2:** Daily maternal milk output ≥ 350 g/d, 500 g/d and 750 g/d (*n*, %).

**Milk output**	**D7**	**D14**	**D21**	**D28**	**D35**	**D42**
≥ 350 g/d	17 (56.7)	28 (93.3)	28 (93.3)	28 (93.3)	28 (93.3)	28 (93.3)
≥ 500 g/d	13 (43.3)	19 (63.3)	24 (80.0)	26 (86.7)	28 (93.3)	28 (93.3)
≥ 750 g/d	5 (16.7)	11 (36.7)	16 (53.3)	19 (63.3)	20 (66.7)	22 (73.3)

**Figure 3:**
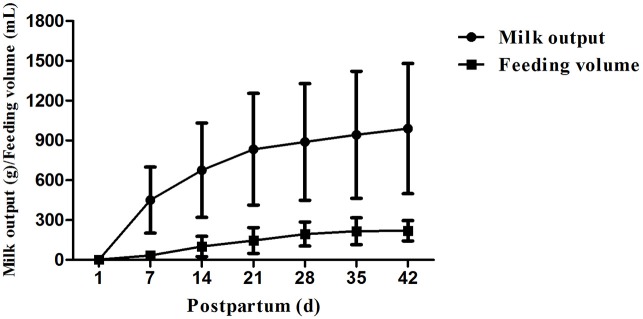
Milk output and feeding volume on days 7, 14, 21, 28, 35 and 42 (all *P* < 0.001).

The mean milk output of mothers of single birth on day 1 was less than the mean feeding volume of their babies (*P* < 0.001, Figure 3). The mean milk output gradually increased and exceeded the mean feeding volume on days 7, 14, 21, 28, 35, and 42 (*P* < 0.001, Figure 3). The percentages of milk output in mothers of single birth exceeding feeding volume of their infants were 43.5% on day 1, 100% on days 7, 14, and 21, 95.6% on days 28 and 35, and 91.3% on day 42, respectively.

The milk output of mothers using hospital-grade breast pumps in this study were compared with that of mothers of VLBW infants with early exclusive hand expression in the literature (8). Except for the first 2 days after birth, mothers using hospital-grade breast pumps in this study had significantly (*P* < 0.05, Table 3) more cumulative daily milk production throughout the first 14 days postpartum compared with mothers using exclusive hand expression in the literature.

**Table 3:** Comparison of mothers with hospital-grade breast pump in this study vs. mothers with hand expression in the literature.

	**This study (hospital-grade breast pump, g, *n* = 30)**	**The literature (hand expression, mL, *n* = 12)**	***Z***	***P*-value**
**Cumulative milk output after**				
D1	0.0 (0.0, 0.0)	0.1 (0.0, 2.6)	0	<0.001
D2	0.0 (0.0, 0.6)	0.25 (0, 11)	201.0	0.493
D3	28.8 (3.0, 82.9)	15 (0.3, 41)	355.0	0.012
D4	173.3 (94.6, 360.5)	119 (6, 167)	371.0	0.004
D5	428.8 (269.7, 828.5)	216 (28, 267)	416.0	<0.001
D6	820.0 (482.3, 1308.8)	359 (59, 594)	440.0	<0.001
D7	1139.3 (827.1, 1289.3)	456 (104, 866)	456.0	<0.001
D14	5802.6 (3630.5, 8406.7)	2,387 (1,196, 3,952)	453.0	<0.001
**Cumulative milk output between**				
D8-14	4560.8 (2866.4, 6646.7)	1,789 (957, 3,320)	453.0	<0.001

### Correlations Between Milk Output on Days 7 and 14 With That on Other Days

Pearson correlation analysis was used to analyze the correlations of milk output on days 7 and 14 with that on other days (Table 4). Milk yield on day 7 was positively correlated to those on days 14, 21, 28, 35, and 42. Milk yield on day 14 also had positive correlations with those on days 21, 28, 35, and 42. As the study progressed, the correlation gradually weakened (day 7 with day 14, *r* = 0.886; day 7 with day 42, *r* = 0.756; day 14 with day 21, *r* = 0.951; day 14 with day 42, *r* = 0.843; all *P* < 0.001).

**Table 4:** Correlations between milk output on days 7 and 14 and other days.

	**D14**		**D21**		**D28**		**D35**		**D42**	
	***r***	***P*-value**	***r***	***P*-value**	***r***	***P*-value**	***r***	***P*-value**	***r***	***P*-value**
D7	0.886	<0.001	0.847	<0.001	0.813	<0.001	0.841	<0.001	0.756	<0.001
D14	1		0.951	<0.001	0.925	<0.001	0.877	<0.001	0.843	<0.001

### Factors Affecting Milk Output

Mothers were assigned to one of two groups based on their mean pumping frequency. On days 7, 14, and 42, the mean milk output of mothers with a pumping frequency of ≥ 6 times/d was greater than that of mothers with a pumping frequency of < 6 times/d. On postpartum day 42, this difference was statistically significant (*t* = −2.828, *P* = 0.009, Table 5). On days 7, 14, and 42, mothers who started pumping within 6 h after delivery (*n* = 12) had the same milk output as those who started pumping after 6 h or more (*n* = 18). The time from delivery to the OOL-II was also associated with milk output. Mothers who reached the OOL-II within 72 h had significantly higher milk output on postpartum days 7, 14, and 42 (*P* < 0.001, Table 5).

**Table 5:** Milk output with different pumping frequency, first pumping time or OOL-II time.

	***n***	**Milk output (g/d)**		
		**D7**	**D14**	**D42**
**Pumping frequency**				
<6 times/d	4	299.5 ± 213.1	447.5 ± 361.3	557.5 ± 308.2
≥ 6 times/d	26	490.1 ± 231.0	768.5 ± 348.2	1178.7 ± 419.4
*t*-test		−1.549	−1.71	−2.828
*P*-value		0.133	0.098	0.009
**First pumping time (h)**				
≤6 h	12	480.3 ± 239.2	794.1 ± 420.9	1096.9 ± 551.4
> 6 h	18	454.2 ± 237.9	680.0 ± 319.6	1095.2 ± 396.8
*t*-test		0.294	0.843	0.010
*P*-value		0.771	0.406	0.992
**Time to reach OOL-II**				
≤72 h	12	654.6 ± 183.0	1028.5 ± 252.1	1436.9 ± 278.4
>72 h	18	344.0 ± 183.7	523.8 ± 270.8	868.6 ± 409.0
*t*-test		4.412	5.137	4.197
*P*-value		<0.001	<0.001	<0.001

The mean milk output of mothers who had multiple births was not significantly different from that of mothers who had single births (*P* > 0.05). On postpartum days 7, 14, and 42, the mean milk output of mothers with GA ≥ 30 weeks vs. < 30 weeks were not significantly different (*P* > 0.05).

### Predictors of Milk Output > 750 g/d and > 1,000 g/d on Day 42

**Figure 4:**
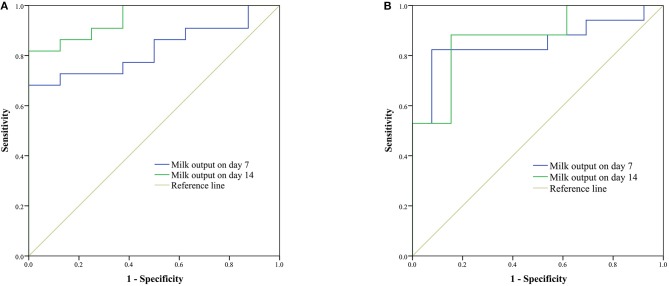
Receiver operating characteristic (ROC) curves of milk output on days 7 and 14 for prediction of milk output > 750 g/d **(A)** and > 1,000 g/d **(B)** on day 42. **(A)** For the cut-off values of 406.8 g/d on day 7 [the area under the curve (AUC) was 0.824; 95% *CI* was 0.678–0.970; *P* = 0.008; sensitivity was 68.2%; specificity was 100%] and 518.0 g/d on day 14 (AUC was 0.949; 95% *CI* was 0.875–1.000; *P* < 0.001; sensitivity was 81.8%; specificity was 100%) were selected to predict the milk output being more than 750 g/d on day 42. **(B)** For the cut-off values of 406.8 g/d on day 7 (AUC was 0.851; 95% *CI* was 0.705–0.996; *P* = 0.001; sensitivity was 82.4%; specificity was 94.3%) and 600.9 g/d on day 14 (AUC was 0.873; 95% *CI* was 0.745–1.000; *P* = 0.001; sensitivity was 88.2%; specificity was 84.6%) were selected to predict the milk output being more than 1,000 g/d on day 42.

As shown in Figure 4, ROC curves were used to analyze the predictive value of milk output on days 7 and 14 for milk output > 750 g/d (Figure 4A) and > 1,000 g/d (Figure 4B) on day 42. Cut-off values of milk output on day 7 (406.8 g/d) and day 14 (518.0 g/d) were identified as predicting milk output of more than 750 g/d on day 42. Cut-off values for milk output on day 7 (406.8 g/d) and day 14 (600.9 g/d) were identified as predicting milk output of more than 1,000 g/d on day 42.

### Milk Composition

A total of 309 samples from 8 participants were analyzed with the Miris HMA. Of these, 168 samples were colostrum, with 56 sets of fore-, mixed-, and hind-milk samples, and 141 samples were transitional milk, with 47 sets of fore-, mixed-, and hind-milk samples.

**Figure 5:**
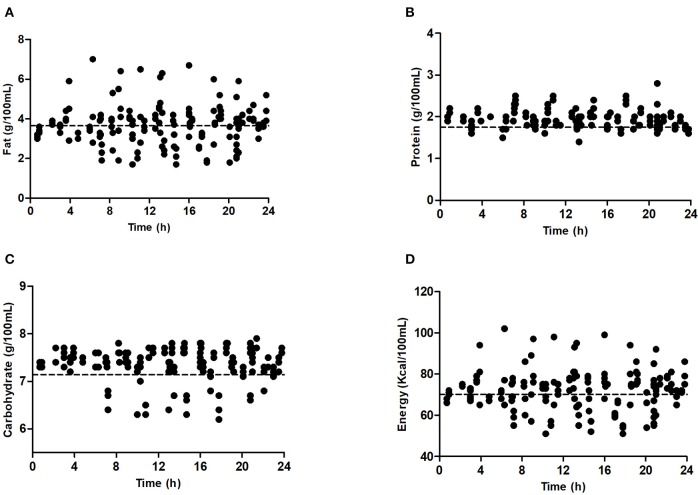
Nutrients in milk sample on day 7. **(A)** Fat; **(B)** protein; **(C)** carbohydrate; **(D)** energy.

Fat and energy levels were higher in hind-milk than fore- or mixed-milk on days 7 and 14 (*P* < 0.001, Table 6). Fat and energy levels varied greatly over 24 h on day 7, but protein and carbohydrate levels remained relatively stable (Figure 5). Variation in nutrients on day 14 showed the same trends.

**Table 6:** Nutrients in human milk on days 7 and 14.

		**Fat (g/100 mL)**	**Protein (g/100 mL)**	**Carbohydrate (g/100 mL)**	**Energy (Kcal/100 mL)**
D7	*n*	56	56	56	56
	Fore-milk	3.31 ± 0.80	1.94 ± 0.19	7.33 ± 0.43	68.43± 7.77
	Mixed-milk	3.62 ± 0.73	1.96 ± 0.21	7.43 ± 0.32	71.77 ± 7.16
	Hind-milk	4.14 ± 1.24	1.94 ± 0.25	7.35 ± 0.43	76.34 ± 11.57
	*F*	10.945	0.115	1.067	10.798
	*P*-value	<0.001	0.892	0.346	<0.001
D14	*n*	47	47	47	47
	Fore-milk	3.6 ± 1.1	1.73 ± 0.16	7.64 ± 0.31	71.47 ± 10.84
	Mixed-milk	4.6 ± 0.9	1.69 ± 0.16	7.61 ± 0.18	80.68 ± 8.78
	Hind-milk	5.2 ± 1.1	1.67 ± 0.18	7.51 ± 0.27	86.49 ± 10.42
	*F*	28.693	1.667	3.391	26.689
	*P*-value	<0.001	0.193	0.036	<0.001

**Figure 6:**
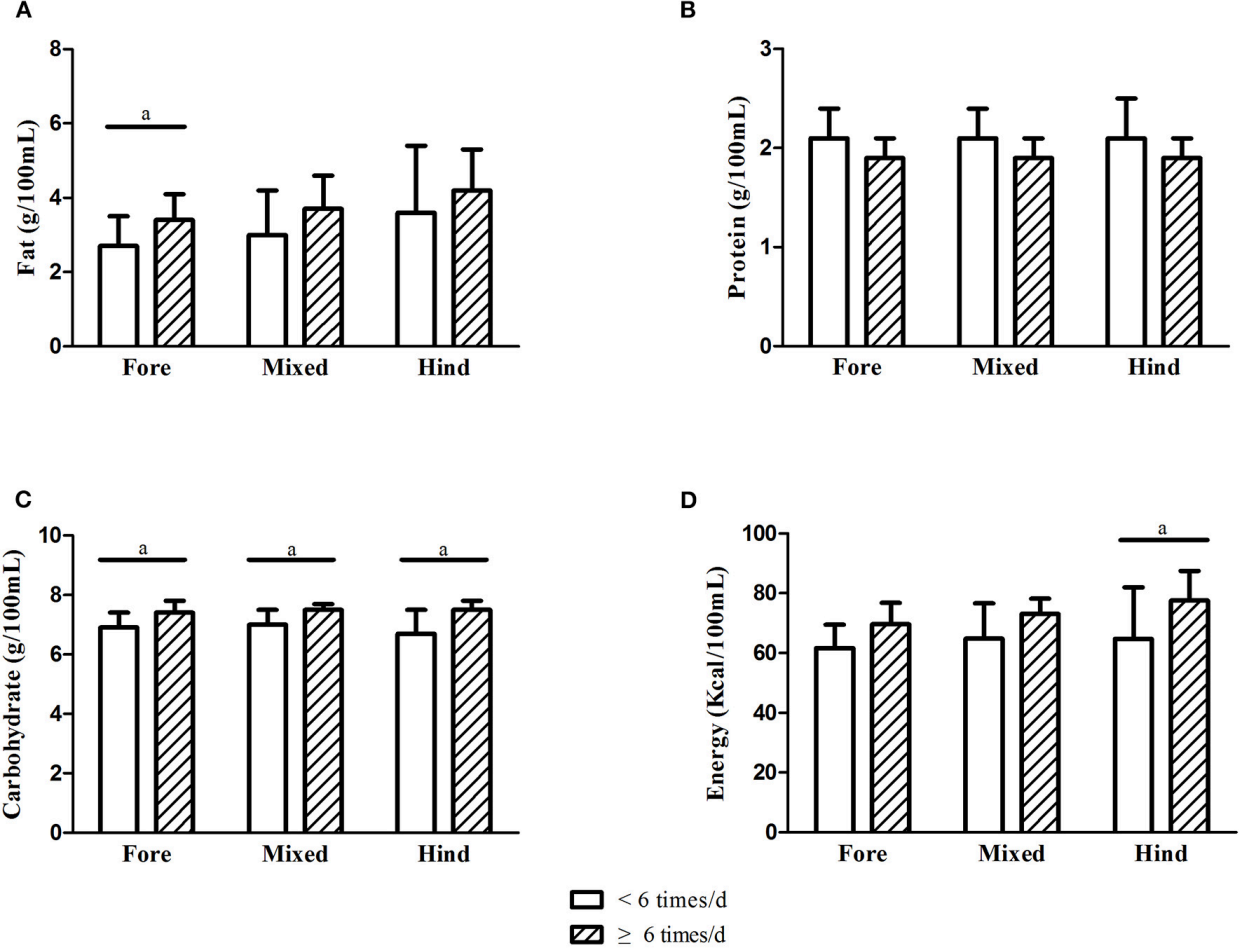
Comparison of nutrients of breastmilk with different pumping frequency on day 7. **(A)** Fat; **(B)** protein; **(C)** carbohydrate; **(D)** energy (a, *P* < 0.05).

Those who pumped 6 times/d or more had higher fat, carbohydrate, and energy levels in their milk on day 7 (Figure 6: A, fat; B, protein; C, carbohydrate; D, energy). However, these differences were no longer present on day 14.

## Discussion

This research was conducted to investigate the breast pumping practices, milk production, milk nutrient content in mothers separated from their preterm infants using hospital-grade breast pumps. These data will be used to establish a baseline for the initiation and maintenance of lactation in mothers of preterm infants, and adds to the limited published literature on the concentration of macronutrients in the milk of mothers of VLBW infants.

In this study, the mean pumping frequency of preterm infants' mothers was 6.7 times per day, with 40% (12/30) of mothers starting pumping within 6 h postpartum, and 83.3% (25/30) of mothers starting pumping within 12 h. The median time from delivery to starting pumping was 7.6 h. Reasons for a delay in pumping (more than 6 h) may include pumps not being available prior to entering the study, insufficient support from family and staff, time-consuming transfers of infants to our hospital, and insufficient preparation for breast feeding sick infants.

The OOL-II typically occurs within 48–72 h after birth. Some researchers defined DOL-II as lactation onset occurring after 72 h postpartum (11, 12). DOL-II was a common issue occurring in mothers of preterm infants (4, 13). The definition of OOL-II in this study is a pragmatic approach similar to that reported by Meier et al. (14). The incidence of DOL-II (60%) in our study was higher than that reported in other Chinese literature (36%) (15), however, the GA and birthweight in our study were lower than that in the Chinese literature. In addition, the mean time to reach the OOL-II in this study (75.4 h) was shorter than that reported in mothers of VLBW infants (97.15 h) (16).

Linked to eventual milk output and duration of lactation, the successful completion of the transition from initiation to maintenance is a critical process (17). Preterm expressing mothers often suffer from inadequate milk supply (4). The minimum milk volume needed by preterm infants were 350 mL/d, 500 mL/d, and 750 mL/d when they are in the NICU, discharged from the NICU, and several months post-birth, respectively (18). These data showed that 56.7% and 43.3% of participants with hospital-grade breast pump produced milk output ≥ 350 g/d and 500 g/d on day 7. This rose to 63.3% on day 14 and 93.3% on day 42, similar to the percentage reported by Post et al. (19) in 2016 (91% in < 34 w group using I-BPSP). With the help of hospital-grade breast pump, mothers had more cumulative daily milk production than using exclusive hand expression in the literature (8). In addition, on days 7, 14, 21, 28, 35, and 42 postpartum, the mean milk output was all greater than the feeding volume of their babies. Furthermore, milk output on day 21 (870.7 ± 423.5 g/d) was greater than that reported by Parker et al. (20) in 2012 for VLBW infants' mothers with early initiation (within 1 h) or late initiation (1–6 h) pump (613.0 mL and 267.2 mL, respectively). In our study, highly positive correlations were found among the week-to-week milk output, similar to previous studies (4, 21). Moreover, milk output on day 7 (area under the curve (AUC) = 0.824; *P* = 0.008) and day 14 (AUC = 0.949; *P* < 0.001) both predicted milk output (> 750 g/d) on day 42 with a high level of significance. These data suggest that milk output ≥ 406.8 g/d on day 7 and ≥ 518.0 g/d on day 14 is the most significant value for predicting milk adequacy on day 42.

Associations have been established by many previous studies between pumping initiation, pumping frequency, and milk output (20, 22). In this study, pumping frequency and milk output on day 42 were positively correlated. Compared with those who pumped <6 times/d, mothers who pumped ≥ 6 times/d produced significantly more milk on day 42 (*P* = 0.009). This suggests that pumping at least 6 times/d can help improve milk output on day 42, which is consistent with the results of previous research (9). The current study did not find significant associations between early initiation of milk expression and milk output, differing from previous studies (8, 20). Possible explanations are that the early-initiation mothers in our study may not have initiated pumping early enough to make a difference, e.g., < 1 h postpartum (20) or that the number of participants in this study was too small. The difference in milk output between the multiple birth group and the non-multiple birth group was not significant. Compared with mothers of infants with GA of < 30 w, the GA ≥ 30 w group did not show significant differences in milk output throughout the postpartum period.

The HMA (Miris) was considered to be a convenient and precise instrument for analysis of the macronutrient levels in breast milk (22). Menjo et al. (23) used an HMA to test fat and protein levels of diluted human milk. They proved four-time dilution to be acceptable. However, others have shown that dilution might affect the performance of the device (24, 25). In our study, we used undiluted breast milk for measurement. We analyzed milk samples collected over 24 h, including fore-, mixed- and hind- milk. This approach helped us more clearly distinguish the differences among milk samples within a single pumping session. Our milk sample collection method was in line with that in the existing literature (26) but differed from that of Valentine et al. (27). In agreement with other research (22), large variations in the fat and energy content of milk were found in this study both within and between mothers of preterm infants on days 7 and 14. Nevertheless, protein and carbohydrate content remained relatively stable. On day 7, a mean milk fat concentration of 3.31 g/100 mL was observed in fore-milk, 3.62 g/100 mL in mixed-milk and 4.14 g/100 mL in hind-milk. On day 14, fat concentration increased to 3.60 g/100 mL in fore-milk, 4.60 g/100 mL in mixed-milk and 5.20 g/100 mL in hind-milk. These results confirm earlier findings that, as lactation progressed and the breast emptied, the fat content of human milk increased (26, 28), and that fat concentrations were consistently higher in hind-milk than in fore-milk samples from the same pumping session (29). However, the degree of increase in fat concentration from fore-milk to hind-milk on day 7 was not as significant as that reported by Saarela et al. (28) in mothers of term infants or that reported by Bishara et al. in mothers of VLBW infants of <28 weeks' gestation (1.7-fold) (30). The same phenomenon appeared in milk energy concentration patterns. For protein and carbohydrate concentrations, only very slight changes were detected among fore-, mixed-, and hind-milk. Milk protein concentration decreased as time went by. The mean protein concentrations on day 7 for fore-milk and hind-milk were both 1.94 g/100 mL, higher than the values reported by Saarela et al. (28) for protein in full-term fore-milk (1.68 g/100 mL) and hind-milk (1.64 g/100 mL) (28).

According to previous studies (26, 31, 32), the degree of breast emptying is the major factor explaining variance in fat concentrations of milk within women. For pump-dependent mothers of preterm infants, the degree of breast emptying could be decided by pumping frequency/interval and emptying the breasts to highly consistent degrees in each pumping session. In this study, we found that those who pumped 6 or more times/d had significantly higher fat, carbohydrate, and energy levels in their fore-milk on day 7.

This was a prospective observational cohort study to assess the initiation of lactation, milk production and milk nutrient content with the help of hospital-grade breast pumps in mothers who were separated from their preterm infants. With the help of a hospital-grade pump, most preterm infants' mothers can start lactation and reach full lactation successfully. The recommended pumping frequency is at least 6 times/d. There is great variation in the nutrient levels of milk from mothers of preterm infants, so nutrient analysis of breast milk is needed. These results are critical for mothers of preterm infants and every NICU workers. Based on our research, we can provide professional lactation support for mothers of preterm infants. Further research involving mothers of VLBW infants is needed to determine early intervention measures that may improve milk output of mothers at risk of lactation failure.

This study had some limitations. The number of participants was small due to the heavy participation requirements of this study. We did not document the specific nutritional habits of the mothers, which might somehow affect milk contents, especially fat content. However, the study was carried out in a tertiary university teaching hospital, all mothers received regular prenatal examinations and detailed nutritional guidance during pregnancy and after delivery. The mother's nutritional status was relatively balanced. In addition, this was an observational cohort, meaning that it was the first step in a longer program of research, a multi-center research will be carried out in the future. As our study was carried out in a tertiary university teaching hospital, it is possible that the conclusions drawn from it may only be applicable to other similar hospitals.

## Data Availability Statement

The raw data supporting the conclusions of this article will be made available by the authors, without undue reservation, to any qualified researcher.

## Ethics Statement

The studies involving human participants were reviewed and approved by the Ethics Committee of Peking University First Hospital. The patients/participants provided their written informed consent to participate in this study.

## Author Contributions

XR, XH, and QF designed the study and analyzed the data. XR and XH acquired the data, organized the database, and drafted the manuscript. QF read and revised the manuscript. All authors contributed to all study data, write and approved the final version of the manuscript.

## Conflict of Interest

The authors declare that the research was conducted in the absence of any commercial or financial relationships that could be construed as a potential conflict of interest.
